# A Systematic Review of Arnica montana in Postoperative Recovery Across Surgical Specialties

**DOI:** 10.7759/cureus.113960

**Published:** 2026-08-04

**Authors:** Emily R Stack, Colby V Spongberg, Kathleen O'Neal, Rachel Rosado

**Affiliations:** 1 Department of Surgery, William Carey University College of Osteopathic Medicine, Hattiesburg, USA; 2 Department of Surgery, Touro College of Osteopathic Medicine, Great Falls, USA; 3 Department of Surgery, Hattiesburg Clinic, Hattiesburg, USA

**Keywords:** arnica montana, complementary therapy, ecchymosis, edema, herbal medicine, pain management, postoperative recovery, randomized controlled trials, surgical specialties, systematic review

## Abstract

*Arnica montana* is a popular herbal supplement used to minimize bruising, swelling, and discomfort after surgery, but evidence of its efficacy remains inconsistent across surgical specialties. This systematic review, conducted according to PRISMA guidelines, searched PubMed, Embase, Cochrane Library, Trip Database, and ClinicalTrials.gov on June 13, 2025, for studies evaluating perioperative *A. montana* (oral or topical) in human surgical patients with reported postoperative outcomes. Risk of bias was assessed using Cochrane RoB 2 for randomized trials, and results were synthesized narratively with subgroup analysis by specialty. Of 266 records identified, 27 studies met inclusion criteria, with plastic surgery accounting for the largest proportion (n = 10), followed by orthopaedics (n = 5), breast surgery, cardiothoracic surgery, hand surgery, otolaryngology, vascular surgery, general surgery, obstetrics/gynecology, and urology. Across all specialties, 55.6% (n = 15) of studies reported measurable benefit from *A. montana*, 33.3% (n = 9) found no significant effect, and 11.1% (n = 3) were ongoing trials without published results. Plastic surgery demonstrated the highest proportion of reported benefit, while evidence in other specialties was mixed. Overall, *A. montana* shows moderate potential as an adjunct in postoperative recovery, particularly in plastic surgery, but inconsistency across trials highlights the need for standardization of dosing, administration route, and outcome measurement. Further high-quality randomized studies are warranted to clarify its role in surgical recovery protocols.

## Introduction and background

Introduction

*Arnica montana (A. montana)* is an herbal supplement used to reduce postoperative bruising, swelling, and pain [1-3]. It is referred to by many names, including leopard's bane, wolf's bane, and mountain tobacco. The herb is native to European mountains and North America, belonging to the Asteraceae family, which includes daisies and sunflowers [4].

Historically, *A. montana* has been used to treat a variety of conditions with anecdotal effects of antibacterial, antitumor, antioxidant, anti-inflammatory, antifungal, and immunomodulatory activity [5]. Despite its long history of medicinal use, conflicting views regarding* A. montana's* efficacy emphasize the need for more rigorous, evidence-based research [6-8]. It is important to note that the herb has been deemed unsafe for use in oral and injectable routes, but has a low risk of adverse effects when used topically in pain relief products [9].

Today, *A. montana* is widely used in homeopathic medicine as a topical herbal medication for inflammation, postoperative bruising, swelling, and pain with promising results [10-14]. The chemical composition of the herb includes sesquiterpene phenolic acids, lactones, flavonoids, essential oils, diterpenes, carotenoids, and oligosaccharides [15-18]. Specifically, the esters of helenalin are deemed responsible for the anti-inflammatory effects of *A. montana* by preventing the activation of nuclear factor kappa B (NF-kB) [17].

An additional factor driving interest in *A. montana* is the broader movement toward opioid-sparing postoperative recovery strategies. Over the past decade, surgical specialties have increasingly emphasized multimodal pain management to reduce reliance on opioid medications while maintaining adequate analgesia. Studies suggest that approximately 3-7% of opioid-naïve surgical patients develop persistent opioid use following surgery, highlighting the importance of identifying safe alternatives and adjunctive therapies that may improve postoperative recovery while minimizing narcotic exposure [19]. Enhanced Recovery After Surgery (ERAS) pathways now incorporate multimodal analgesia strategies, including nonsteroidal anti-inflammatory drugs (NSAIDs), acetaminophen, regional anesthesia, and complementary therapies to optimize recovery and reduce opioid requirements [20-22]. Within this evolving framework, natural adjuncts, such as* A. montana*, have gained attention due to their reported anti-inflammatory and analgesic properties. Although *Arnica *is commonly recommended in plastic and cosmetic surgery practices to reduce bruising and swelling, its potential role within modern multimodal postoperative care pathways has not been systematically evaluated across surgical specialties.

Despite its popularity, clinical evidence for its efficacy across surgical specialties remains inconsistent. A systematic evaluation of its perioperative role is warranted, given growing patient interest in natural recovery adjuncts. This review aims to synthesize evidence from human surgical studies assessing *A. montana's *effect on postoperative outcomes, including pain, edema, ecchymosis, hematoma formation, and overall recovery. Furthermore, *A. montana *preparations used clinically are not uniform. Herbal preparations typically contain measurable amounts of biologically active compounds and are most commonly administered topically, whereas homeopathic preparations are highly diluted according to homeopathic principles and may contain little or no detectable active ingredient. Because these formulations differ substantially in composition and proposed mechanisms of action, their efficacy should not be assumed to be equivalent. Both types of preparations were represented among the studies included in this review.

This article was presented previously as an abstract at the 2025 American College of Osteopathic Surgeons Annual Clinical Assembly on September 18, 2025, in Salt Lake City, UT.

Methods

This systematic review follows the guidelines of Preferred Reporting Items for Systematic Reviews and Meta-Analyses (PRISMA). The review protocol was registered on the International Prospective Register of Systematic Reviews (PROSPERO) on September 9, 2025, and is available online (CRD420251143565).

Eligibility Criteria

Studies evaluating the use of *A. montana *for perioperative or postoperative recovery in human surgical patients were eligible for inclusion. Eligible procedures included any surgical specialty in which *A. montana* was administered orally or topically to improve postoperative outcomes such as pain, edema, ecchymosis, hematoma formation, or overall recovery. Included studies evaluated a variety of Arnica preparations, including topical herbal formulations, homeopathic preparations of varying dilutions, and combination products containing *Arnica *with other active ingredients. Studies had to provide measurable outcomes, either quantitative or qualitative, sufficient for descriptive analysis. Eligible participants were adults. Searches were limited to human studies published in English. No publication date restrictions were applied.

Studies were excluded if they were reviews, editorials, or commentaries, if they lacked human participants, or if they reported irrelevant or non-comparable outcomes. Studies without available outcome data were also excluded.

Information Sources and Search Strategy

A systematic search was used to identify studies utilizing the MEDLINE (Ovid, New York, NY), Embase (Elsevier, Amsterdam, The Netherlands), the Cochrane Central Register of Controlled Trials (Wiley, Hoboken, NJ), ClinicalTrials.gov (National Library of Medicine, Bethesda, MD), and Trip Database (Cardiff, UK). Controlled vocabulary terms were used when available. The search was conducted on June 12, 2025, using terms related to "*Arnica montana*" and "surgery" with database-specific search strategies listed in Table 1.

**Table 1:** Search terms Database-specific search strategy and keywords used across databases for the identification of studies evaluating *Arnica montana *in postoperative surgical recovery.

Database	Keywords/Free Text
MEDLINE (Ovid) (73 records)	“Arnica montana” OR Arnica OR “mountain tobacco” OR “wolf’s bane” OR “leopard’s bane”; surgery OR surgical OR operative OR perioperative OR postoperative OR “plastic surgery” OR rhinoplasty OR blepharoplasty OR mastectomy OR orthopaedic OR “knee surgery” OR tonsillectomy OR hysterectomy
Embase (Elsevier) (170 records)	“Arnica montana” OR Arnica; surgery OR operative OR perioperative OR postoperative OR rhinoplasty OR blepharoplasty OR breast surgery OR orthopaedics OR vascular surgery OR gynecology OR urology
Cochrane Central Register of Controlled Trials (4 records)	“Arnica montana” OR Arnica; surgery OR postoperative OR perioperative OR operative OR rhinoplasty OR blepharoplasty OR tonsillectomy OR mastectomy
ClinicalTrials.gov (3 records)	Intervention: Arnica
Trip Database (16 records)	“Arnica montana” AND (surgery OR postoperative OR perioperative OR rhinoplasty OR blepharoplasty OR orthopaedic OR mastectomy OR tonsillectomy OR hysterectomy)

Study Selection

Two reviewers (ERS and KO) independently screened titles and abstracts using predefined inclusion and exclusion criteria. Full-text articles were then reviewed for final eligibility. Discrepancies were resolved by discussion. Screening was managed using the Rayyan AI-Powered Systematic Review Management Platform (Cambridge, MA).

Data Collection Process and Data Items

Two reviewers (ERS and KO) independently extracted data from each included study using a standardized, pre-specified data collection form developed prior to full-text review. Extracted variables included study title, year of publication, authors, country of origin, surgical specialty, procedure type, study design, sample size, route and timing of *A. montana *administration (oral or topical), dosing regimen, comparator or control intervention, outcome measures assessed, and reported postoperative outcomes.

Primary outcomes of interest included postoperative pain, edema, ecchymosis, hematoma formation, and overall recovery measures. Secondary outcomes included complications, adverse events, and patient-reported satisfaction when available. Data extraction was performed independently by both reviewers to minimize extraction bias. After extraction, results were compared, and discrepancies were resolved through discussion and consensus. When needed, a third reviewer was consulted to resolve disagreements.

Extracted data were compiled into a master spreadsheet using Microsoft Excel (Microsoft Corporation, Redmond, WA), which was subsequently used to generate descriptive summaries and graphical representations of study characteristics and outcomes.

Statistical Analysis

A quantitative meta-analysis was not performed due to heterogeneity among studies, including differences in surgical specialties, *A. montana* formulations, dosing, timing of administration, outcome definitions, follow-up duration, and study designs. Consequently, statistical pooling was considered inappropriate for this review.

Results were therefore synthesized using a structured narrative approach. Studies were grouped by surgical specialty and categorized according to whether they demonstrated a statistically significant improvement in one or more predefined postoperative outcomes (pain, edema, ecchymosis, hematoma formation, or overall recovery) compared with the control group. Studies reporting no statistically significant improvement were categorized as "no benefit." Graphs illustrating study characteristics, specialty distribution, and reported effectiveness were generated in Microsoft Excel (Redmond, WA). Ongoing clinical trials without published outcome data were summarized separately. No pooled effect estimates, confidence intervals, heterogeneity statistics, or meta-regression analyses were performed.

Risk of Bias Assessment

**Figure 1:**
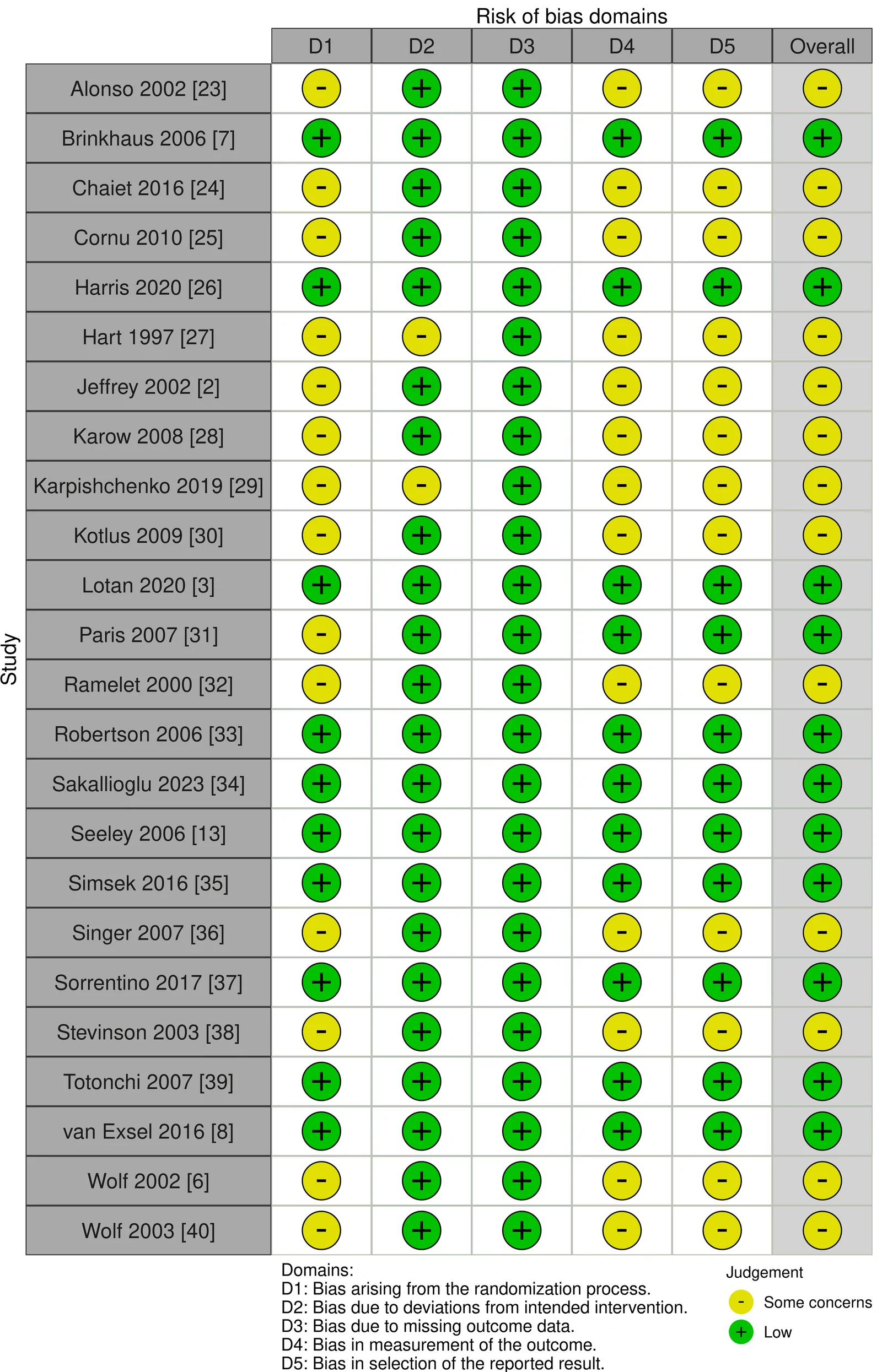
Risk of bias assessment Randomized controlled trials underwent risk of bias assessment [2,3,6-8,13,23-40]. Risk of bias assessment of included randomized controlled trials using the Cochrane Risk of Bias 2 (RoB 2) tool across five methodological domains: randomization process, deviations from intended interventions, missing outcome data, measurement of outcomes, and selection of reported results [41,42].

Risk of bias for randomized controlled trials was assessed using the Cochrane Risk of Bias 2 (RoB 2) tool. This instrument evaluates potential bias across five methodological domains: (D1) bias arising from the randomization process, (D2) bias due to deviations from intended interventions, (D3) bias due to missing outcome data, (D4) bias in measurement of outcomes, and (D5) bias in selection of the reported result (Figure 1).

Two reviewers (ERS and KO) independently performed the risk of bias assessments for each randomized study. Each domain was classified as "low risk," "some concerns," or "high risk" of bias according to the RoB 2 guidance. An overall risk-of-bias judgment was then assigned to each study based on the domain-level assessments.

Any disagreements between reviewers were resolved through discussion. When consensus could not be reached, the study was re-evaluated jointly to ensure consistency with Cochrane methodological guidance. The results of the risk-of-bias assessment were summarized qualitatively and considered during the interpretation of the study findings.

## Review

Results

**Figure 2:**
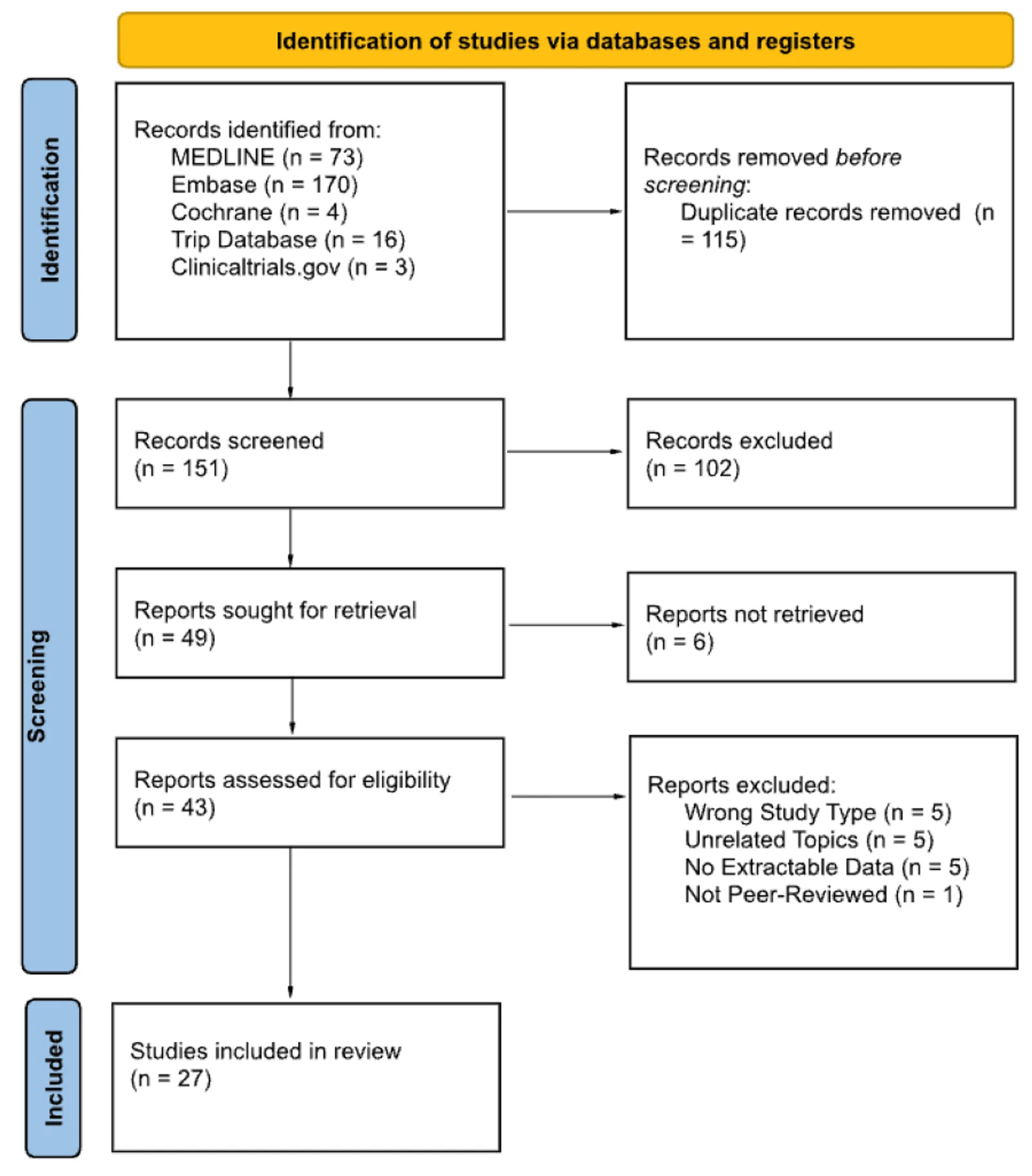
PRISMA flow diagram of study selection A total of 266 records were identified from five databases. After removal of 115 duplicates and screening of 151 records, 27 studies met the final eligibility criteria and were included in the systematic review.

The search yielded 266 records, of which 115 duplicates were removed prior to screening. After title and abstract review, 151 studies were screened, with 102 excluded. Forty-nine reports were sought for retrieval; six were unavailable. Forty-three studies underwent full-text review, and 27 met the inclusion criteria (Figure 2).

The 27 included studies represented nine surgical specialties. Plastic surgery accounted for the largest proportion of studies (n = 11) [8,13,23,24,26,30,34,35,39,43,44], followed by orthopaedics (n = 5) [7,28,31,36,40]. The remaining studies included breast surgery (n = 2) [3,37], cardiothoracic surgery (n = 1) [25], hand surgery (n = 2) [2,38], general surgery (n = 1) [45], obstetrics/gynecology (n = 1) [27], otolaryngology (n = 2) [29,33], and vascular surgery (n = 2) [6,32] (Table 2).

**Table 2:** Summary of the included studies evaluating Arnica montana in postoperative recovery across surgical specialties

Author	Location	Year	Sample size	Article Title	Procedure	Surgical Specialty	*Arnica* Benefit
Alonso et al. [23]	Miami, FL	2002	19	Effects of Topical Arnica Gel on Post-laser Treatment Bruises	Post-laser	Plastic Surgery	No
Brinkhaus et al. [7]	Berlin, Germany	2006	227	Homeopathic Arnica Therapy in Patients Receiving Knee Surgery: Results of Three Randomised Double-Blind Trials	Knee Surgery	Orthopaedics	Yes
Chaiet & Marcus [24]	Madison, WI	2016	22	Perioperative *Arnica montana *for Reduction of Ecchymosis in Rhinoplasty Surgery	Rhinoplasty	Plastic Surgery	Yes
Cornu et al. [25]	Bron Cedex, France	2010	92	No Effect of a Homoeopathic Combination of Arnica montana and Bryonia alba on Bleeding, Inflammation, and Ischaemia After Aortic Valve Surgery	Aortic Valve Surgery	Cardiothoracic Surgery	No
Harris & Darby [26]	Ontario, Canada	2020	300	Enhanced Recovery after Abdominoplasty Using Perisurgical Nutritional Supplementation	Abdominoplasty	Plastic Surgery	Yes
Hart et al. [27]	Southampton, England	1997	73	Double-Blind, Placebo-Controlled, Randomized Clinical Trial of Homoeopathic Arnica C30 for Pain and Infection After Total Abdominal Hysterectomy	Total Abdominal Hysterectomy	OBGYN	No
Jeffrey & Belcher [2]	West Sussex, England	2002	37	Use of Arnica to Relieve Pain After Carpal-Tunnel Release Surgery	Carpal-Tunnel Release	Hand Surgery	Yes
Kang et al. [43]	Chicago, IL, USA	2021	N/A	Assessing the Effectiveness of Arnica montana and Rhododendron tomentosum (Ledum palustre) in the Reduction of Ecchymosis and Edema After Oculofacial Surgery: Preliminary Results	Oculofacial Surgery	Plastic Surgery	Ongoing
Karow et al. [28]	Frankfurt, Germany	2008	88	Efficacy of Arnica montana D4 for Healing of Wounds After Hallux Valgus Surgery Compared to Diclofenac	Hallux Valgus Surgery	Orthopaedics	No
Karpishchenko et al. [29]	Saint-Petersburg, Russia	2019	43	Actual Considerations of Post-tonsillectomy Case Management	Tonsillectomy	Otolaryngology	Yes
Klinieken [44]	The Netherlands	2025	136	Efficacy of Arnica D1 Ointment After Upper Blepharoplasty: A Randomized, Double-Blind, Placebo-Controlled Study	Upper Blepharoplasty	Plastic Surgery	Ongoing
Kotlus et al. [30]	Tucson, AZ	2009	30	Evaluation of Homeopathic Arnica montana for Ecchymosis After Upper Blepharoplasty: A Placebo-Controlled, Randomized, Double-Blind Study	Blepharoplasty	Plastic Surgery	No
Lotan et al. [3]	Jerusalem, Israel	2020	55	Arnica Montana and Bellis Perennis for Seroma Reduction Following Mastectomy and Immediate Breast Reconstruction: Prospective, Randomized, Double-blinded, Placebo-controlled Trial	Mastectomy + Reconstruction	Breast Surgery	Yes
Clinical Trial [45]	Italy	2010	N/A	A Double-Blind, Randomized Trial of Arnica Comp. Heel Treatment in Patients Undergoing Hemorrhoidectomy in Day Surgery: Evaluation of Postoperative Pain	Hemorrhoidectomy	General Surgery	Ongoing
Paris et al. [31]	Grenoble, France	2007	158	Effect of Homeopathy on Analgesic Intake Following Knee Ligament Reconstruction: A Phase III Monocentre Randomized Placebo-Controlled Study	ACL Repair	Orthopaedics	No
Ramelet et al. [32]	Caen, France	2000	130	Homoeopathic Arnica in Postoperative Haematomas: A Double-Blind Study	Saphenous Stripping	Vascular Surgery	No
Robertson et al. [33]	Cardiff, UK	2006	190	Homeopathic Arnica montana for Post-tonsillectomy Analgesia: A Randomised Placebo Control Trial	Tonsillectomy	Otolaryngology	Yes
Yildirim et. al [34]	Elazığ, Turkey	2023	60	Effect of a Combination of Bromelain and Arnica on Periorbital Edema and Ecchymosis in Septorhinoplasty	Septorhinoplasty	Plastic Surgery	Yes
Seeley et al. [13]	San Francisco, CA, USA	2006	29	Effect of Homeopathic Arnica montana on Bruising in Face-lifts: Results of a Randomized, Double-blind, Placebo-Controlled Clinical Trial	Face Lift	Plastic Surgery	Yes
Simsek et al. [35]	Kirikkale, Turkey	2016	108	Topical Application of Arnica and Mucopolysaccharide Polysulfate Attenuates Periorbital Edema and Ecchymosis in Open Rhinoplasty: A Randomized Controlled Clinical Study	Rhinoplasty	Plastic Surgery	Yes
Singer et al. [36]	Jerusalem, Israel	2007	30	Traumeel S® for Pain Relief Following Hallux Valgus Surgery: A Randomized Controlled Trial	Hallux Valgus Surgery	Orthopaedics	Yes
Sorrentino et al. [37]	Milan, Italy	2017	53	Is There a Role for Homeopathy in Breast Cancer Surgery? A First Randomized Clinical Trial on Treatment With Arnica montana to Reduce Post-operative Seroma and Bleeding in Patients Undergoing Total Mastectomy	Total Mastectomy	Breast Surgery	Yes
Stevinson et al. [38]	Exeter, UK	2003	62	Homeopathic Arnica for Prevention of Pain and Bruising: Randomized Placebo-Controlled Trial in Hand Surgery	Hand Surgery	Hand Surgery	No
Totonchi & Guyuron [39]	Cleveland, OH, USA	2007	48	Randomized Comparison Between *Arnica *and Steroids for Post-rhinoplasty Ecchymosis and Edema	Rhinoplasty	Plastic Surgery	Yes
van Exsel et al. [8]	Zeist, The Netherlands	2016	136	Arnica Ointment 10% Does Not Improve Upper Blepharoplasty Outcome: A Randomized, Placebo-Controlled Trial	Blepharoplasty	Plastic Surgery	No
Wolf et al. [6]	Karow, Germany	2003	60	Efficacy of Arnica in Varicose Vein Surgery: Results of a Randomized, Double-Blind, Placebo-Controlled Pilot Study	Varicose Vein Surgery	Vascular Surgery	Yes
Wolf & Rose [40]	Germany	2002	40	Adjuvant Arnica Medication for Swelling and Pain Reduction in Surgically Treated Hip Fractures - Controlled, Non-randomized Therapy Study	Proximal Hip Fracture repair	Orthopaedics	Yes

Of the studies with available outcomes, 62.5% reported a clinical benefit from* A. montana *use. Plastic surgery accounted for the largest proportion of positive findings, with six of nine completed studies demonstrating significant reductions in bruising, swelling, or pain. Orthopaedic studies showed mixed results, with three of five reporting benefits. In specialties with fewer publications, most studies favored *A. montana*, although sample sizes were small.

**Figure 3:**
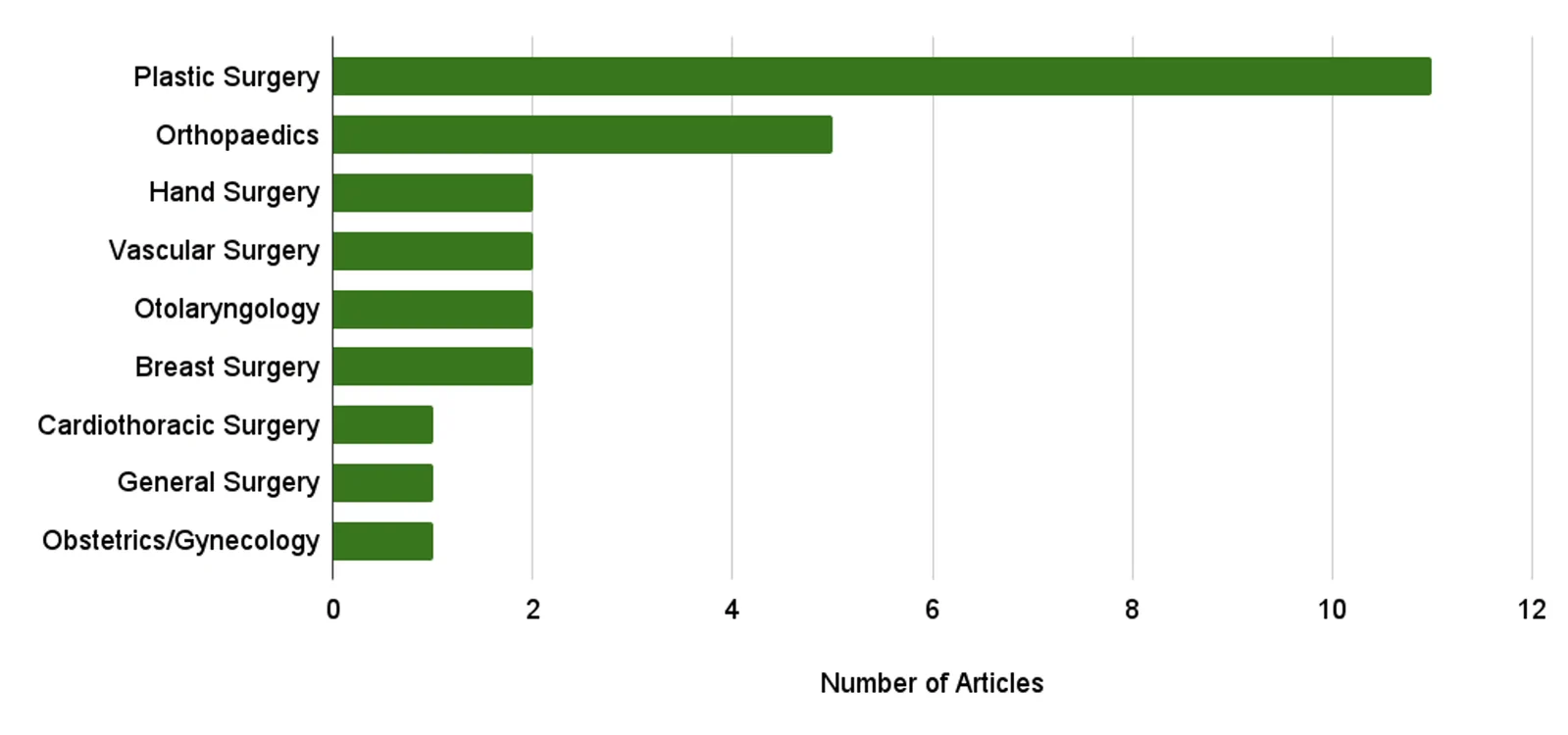
Distribution of the included studies by surgical specialty Plastic surgery accounted for the largest number of publications (n = 11), followed by orthopaedics (n = 5), with fewer studies in vascular, breast, hand, otolaryngology, cardiothoracic, obstetrics/gynecology, and general surgery [2,3,6-8,13,23-30,31-40,43-45].

**Figure 4:**
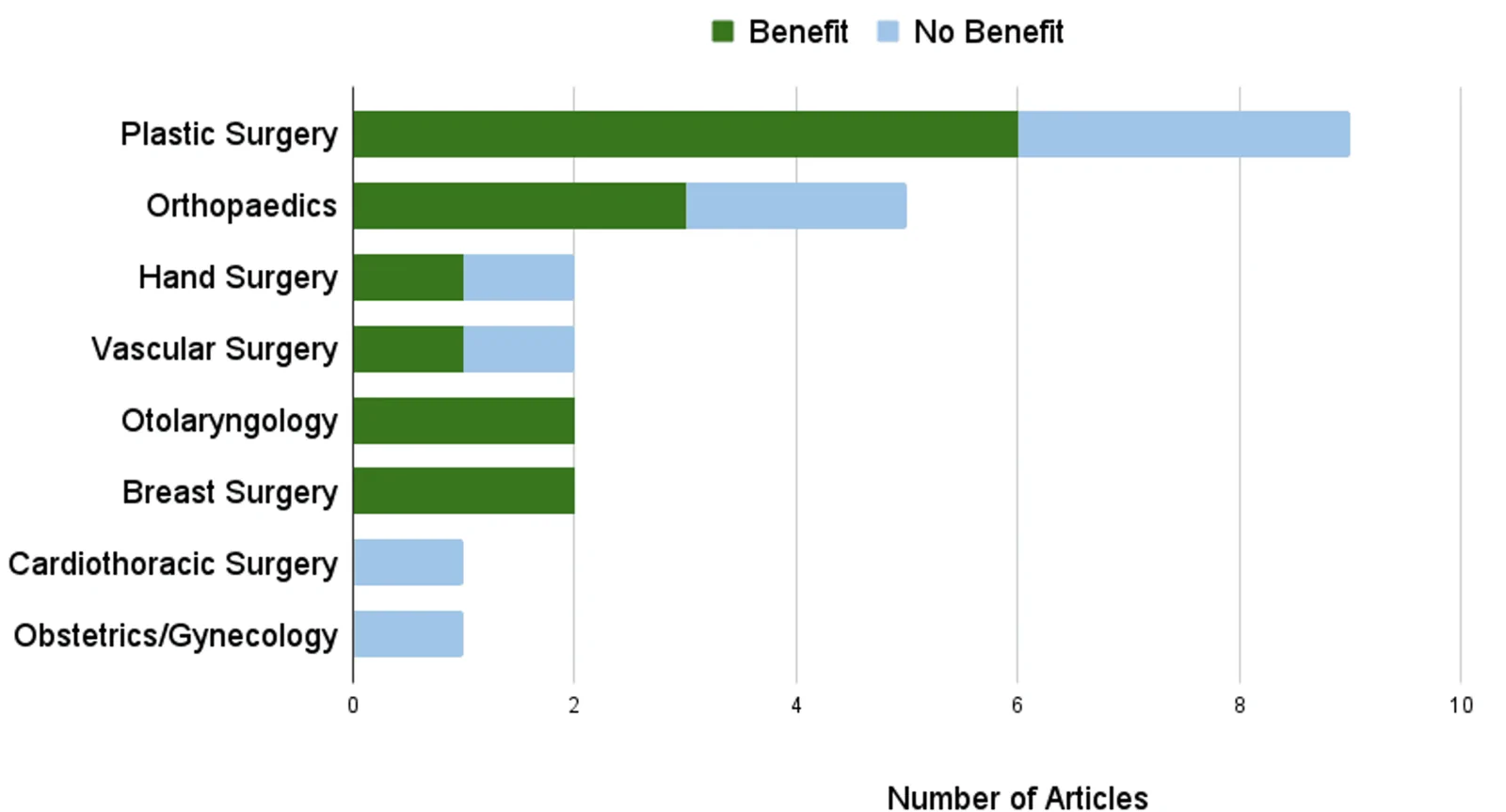
Reported outcomes of Arnica montana use by surgical specialty Plastic surgery demonstrated the greatest number of studies with reported benefit, while orthopaedics and smaller subspecialties showed mixed results [2,3,6-8,13,23-30,31-40,43-45].

**Figure 5:**
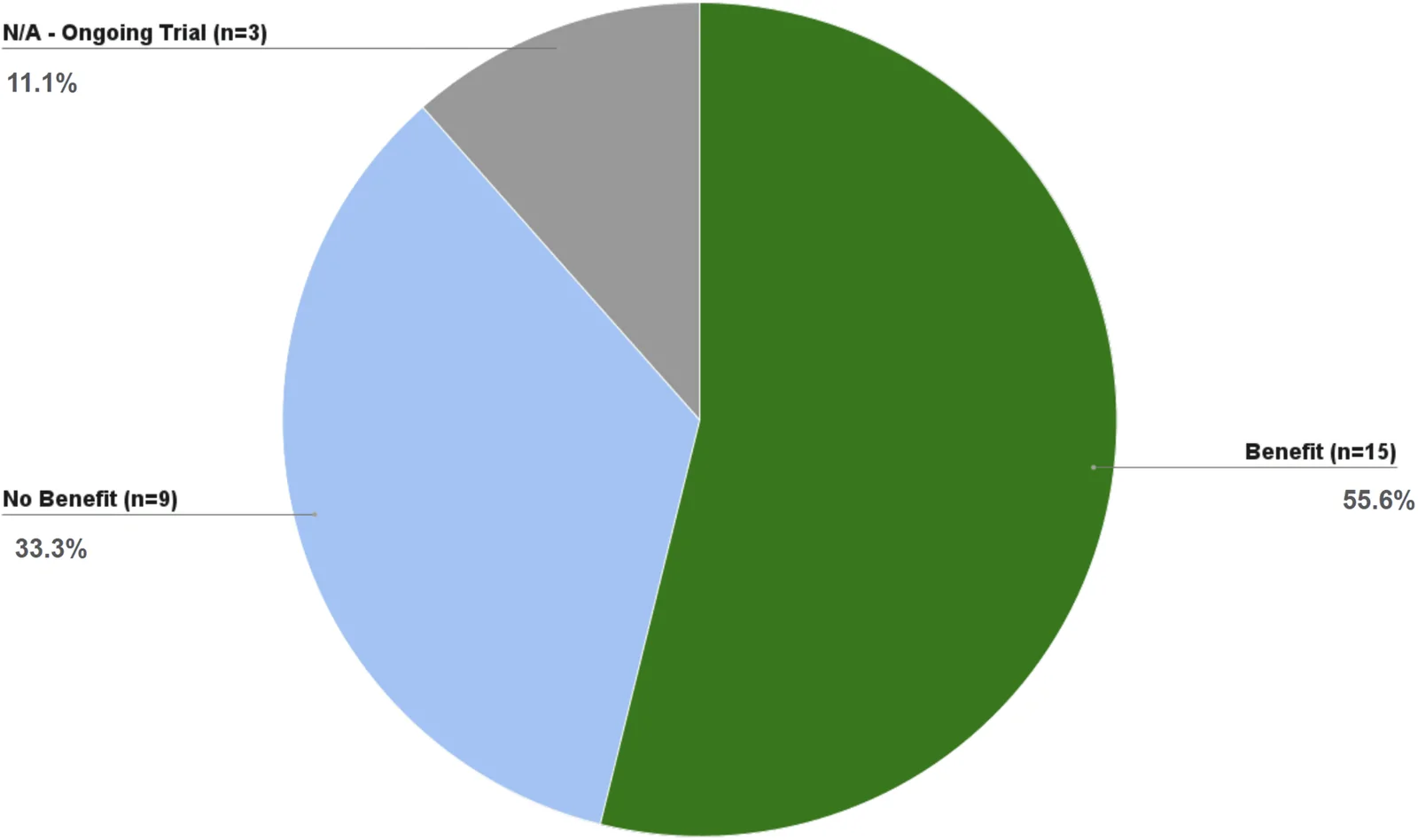
Overall effectiveness of Arnica montana across all included studies Of the 27 studies, 55.6% (n = 15) reported benefit, 33.3% (n = 9) reported no benefit, and 11.1% (n = 3) were ongoing trials without published results [2,3,6-8,13,23-30,31-40,43-45].

Overall, 53.8% of all included studies found benefit, 34.6% reported no significant effect, and 11.5% were ongoing trials without published outcomes (Figures 3-5).

Discussion

This systematic review evaluated the perioperative use of *A. montana *across a broad spectrum of surgical specialties. Unlike prior reviews that focused narrowly on individual fields, such as rhinoplasty or orthopaedics, our analysis integrated data from 30 studies spanning 10 different specialties, providing a more comprehensive view of its clinical potential. Our findings suggest that *A. montana *has moderate evidence supporting its role as an adjunct to postoperative recovery, with 53% of studies demonstrating benefit. The strongest evidence came from plastic surgery, where reductions in bruising, swelling, and pain were consistently reported [13,24,30,34]. Orthopaedic studies showed mixed results, possibly reflecting differences in outcome measures and surgical trauma [7,28,31,36,40]. Evidence from breast, vascular, and other specialties was encouraging but limited by small sample sizes [2,13,23,38,45]. Our findings are generally consistent with those of Gaertner et al., who also reported mixed evidence regarding the effectiveness of *A. montana *for postoperative recovery [11]. While previous reviews focused primarily on selected procedures or individual outcomes, the present review expands upon this work by evaluating *Arnica *use across multiple surgical specialties and incorporating more recent clinical studies.

Role in Multimodal Pain Management

An important contextual consideration in evaluating *A. montana* is the ongoing effort to reduce opioid prescribing in postoperative care. Surgical prescribing practices have historically contributed to excess opioid availability, with substantial variability in both dosage and duration of prescriptions across procedures [46]. In response, many institutions have adopted multimodal analgesia protocols aimed at improving pain control while minimizing opioid exposure. These strategies frequently combine pharmacologic approaches, such as NSAIDs, acetaminophen, and regional anesthesia, with adjunctive therapies designed to reduce inflammation and improve postoperative comfort.

Several studies included in this review reported improvements in postoperative pain scores or reductions in analgesic use among patients receiving *A. montana *[2,7,26,36,37]. Trials in hand surgery and otolaryngology demonstrated improved postoperative comfort and reduced pain following procedures such as carpal tunnel release and tonsillectomy [2,29,33]. Similarly, some orthopaedic studies reported decreased postoperative discomfort after knee and foot surgeries when *Arnica* was administered perioperatively [7,36,40]. Although these findings were not universally consistent, they suggest that *Arnica *may contribute to symptom control during the early inflammatory phase of surgical recovery.

In plastic surgery in particular, postoperative discomfort is often closely associated with tissue edema, ecchymosis, and local inflammatory response rather than deep nociceptive pain. *Arnica'*s proposed anti-inflammatory mechanism through inhibition of nuclear factor kappa B signaling may therefore provide indirect analgesic benefit by reducing the inflammatory processes that contribute to swelling and bruising. By improving these recovery parameters, *Arnica* may enhance patient comfort and satisfaction while potentially decreasing reliance on narcotic analgesics.

However, current evidence remains insufficient to determine whether *A. montana* directly reduces opioid consumption following surgery. Many trials included in this review did not report standardized pain scores, opioid usage, or validated recovery metrics. Future studies evaluating *A. montana* should incorporate objective measures, such as analgesic consumption, validated pain scales, and standardized perioperative protocols. Such data would allow investigators to determine whether Arnica may meaningfully contribute to modern opioid-sparing recovery pathways.

Safety

Safety considerations surrounding *A. montana* remain an important area for future study. While topical preparations are generally regarded as low risk, questions remain regarding systemic use and the potential for adverse effects, such as increased bleeding and bruising. Reports suggesting that higher or repeated doses may shift the balance from therapeutic to harmful highlight the need for standardized formulations, clear dosing protocols, and careful timing of administration in the perioperative setting [47]. Well-designed clinical trials are needed to establish safe and effective dosing regimens tailored to specific surgical contexts [44,47].

Many individuals and medical providers are moving away from traditional forms of narcotics and traditional pain medications. Our study provides evidence that alternatives, such as *A. montana*, provide relief in the form of reduced swelling and pain in many post-operative patients.

Limitations

The main limitations of this review include small study populations, short follow-up periods, and inconsistent methodology among included trials. Differences among herbal, homeopathic, and combination formulations likely contributed to the observed heterogeneity and should be considered when interpreting the results. Many studies relied on subjective endpoints rather than objective measures, such as volumetric edema or quantified ecchymosis. Potential publication bias also cannot be excluded, as studies with neutral findings may be underreported.

## Conclusions

*A. montana* demonstrates potential as a safe adjunct in postoperative recovery, particularly in plastic surgery, where benefits such as reduced edema, ecchymosis, and pain were most consistently reported. However, evidence across other specialties remains mixed, limited by small study sizes, heterogeneous dosing regimens, and reliance on subjective outcome measures. Given the growing demand for non-opioid and natural recovery adjuncts,* A. montana *may represent a useful complementary option in select surgical contexts. However, without standardized formulations, dosing protocols, and validated endpoints, its clinical role cannot be firmly established.

Future research is needed on the subject and should prioritize well-designed, randomized controlled trials across multiple surgical specialties. Studies incorporating objective outcome measures, such as volumetric edema assessment, standardized pain scales, and colorimetric analysis of bruising, will be essential in determining whether *A. montana *provides reproducible benefit beyond placebo. Until such data are available, *A. montana* should be regarded as a potentially helpful, but not yet fully validated, adjunctive therapy in postoperative care.
